# Factors associated with low birth weight among babies born at Hawassa University Comprehensive Specialized Hospital, Hawassa, Ethiopia

**DOI:** 10.1186/s13052-019-0637-7

**Published:** 2019-04-11

**Authors:** Melese Siyoum, Teshome Melese

**Affiliations:** Department of Midwifery, College of Medicine and Health Science, Hawassa University, Hawassa, Ethiopia

**Keywords:** Birth weight, pregnancy, Ethiopia

## Abstract

**Background:**

Low birth weight is defined as infant born with weight of less than 2500 g. It is one of the major public health problems worldwide. In Ethiopia, there are limited evidences on factors contributing to low birthweight.

**Objective:**

To assess factors associated with low birth weight babies in Hawassa University Comprehensive Specialized Hospital, Hawassa, Ethiopia from March to April, 2018.

**Methods and Materials:**

An unmatched case control study was conducted at Hawassa University Comprehensive Specialized Hospital. All low birth weight newborns and two unmatched controls for each case were included in the study from March to April, 2018. Data were collected through face to face interview using a structured and pre-tested questionnaire. The collected data were managed with Epi-data version 3.1 software and exported to the Statistical Package for Social Science (SPSS) version 22. Bivariate and multivariate binary logistic regression were used to identify factors associated with low birth weight at p-value < 0.05 with their respective odds ratios and 95% confidence interval. Hosmer-Lemeshow test was used to assess goodness-of-fit.

**Results:**

In this study 330 mother-newborn pairs (110 cases and 220 controls) were participated making 100% response rate. Among the participants 325(98.48%) were married, 164 (49.7%) were Protestant, 296 (89.7%) had ANC follow up and 212 (64.24%) were multipara. Mothers’ mid-upper arm circumference less than 220 mm [(AOR) =2.89**,** 95% CI**:** 1.58, 5.29**)],** lack of nutritional counseling [AOR = 2.37**,** 95%CI**:** 1.3, 4.34**]**, presence of complications during pregnancy [AOR = 2.96, 95%CI: 1.55, 5.64)] and lack of iron supplementations during pregnancy [AOR = 2.89, 95%CI**:** 1.58, 5.29**)]** were significantly associated with Low birth weight.

**Conclusions:**

Mothers’ mid-upper arm circumference less than 220 mm, lack of nutritional counseling, presence of complications and lack of iron supplementations during current pregnancy were significantly associated with low birth weight. Counseling on nutrition during prenatal care needs attention of service providers.

## Introduction

The World Health Organization (WHO) defines low birth weight (LBW) as a birth weight of infant of 2499 gram or less regardless of gestational age [1]. Low birth weight is further classified into three categories: moderately low birth weight (1500-2499 grams), very low birth weight (VLBW), less than 1500 grams, and extremely low birth weight (ELBW), less than 1000 grams [2].

Overall, it is estimated that 15% to 20% of all births worldwide are LBW, representing more than 20 million births a year [3]. There is considerable variation in the prevalence of low birth weight across regions with estimates of LBW include 28% in south Asia, 13% in sub-Saharan Africa and 9% in Latin America [4].

In Ethiopia, different studies showed variable prevalence of low birth weight. The variations were further reflected in the Ethiopia Demographic Household Survey with the highest rates in 2005 (14%) and lowest in 2011 (11%), although the trend reverts to 13.1% in 2016 report [5]. Similarly, the prevalence of LBW was 16.5% in rural Sidama zone [6], 17.9% in Southwestern Ethiopia [7], 14.6% in Tigray region [8] and 9.1% in Arsi zone [9]. Two studies conducted in Gondar University Hospital indicated that this prevalence ranges from 11.2% to 17.4% [10, 11].

Different studies identified different factors associated with low birthweight. In Laelay Maichew districts, sex of neonate, less than four antenatal care follow ups, unwanted and unplanned pregnancy, and maternal dietary intake per 24 hours during pregnancy were associated with LBW [14]. Pregnancy induced hypertension, malaria during pregnancy, female infant and gestational age less than 37 weeks were identified as the major risk factors for low birth weight in Gondar University Hospital [10, 11]. Low monthly income, lifestyle, and demographic area were another factors identified in south western Ethiopia [12].

Other risk factors, such as maternal age of less than 20 years, mothers with a history of abortion, lack of formal education, residing in rural areas, maternal body mass index less than 18 kg/m2, absence of antenatal care, history of chat chewing, maternal anemia, malnutrition, poor nutrition both before and during pregnancy, extra meal during pregnancy, and lack of iron/folic acid supplementation during pregnancy were all associated with LBW [6, 8, 13–18]. Despite these varied magnitudes and factors affecting fetal birth weight, there is no published data from Hawassa University Comprehensive Specialized Hospital where more than 18 million people receive health services. Since risk factors are vary across settings, the current study was designed to identify factors associated with LBW among babies born at Hawassa University Comprehensive Specialized Hospital, southern Ethiopia.

## Materials and Methods

### Study design and setting

An institutionally based case-control study was conducted at Hawassa University Comprehensive Specialized Hospital (HUCSH) from March to April 25, 2018. Hawassa city is located 273 km south of Addis Ababa, Ethiopia. Currently, Hawassa University Comprehensive Specialized Hospital provides health services for more than 18 million people; and the average number of deliveries per month was around deliveries.

### Populations

All post-partum mother-newborn pairs who visited Hawassa University Comprehensive Specialized Hospital were the source population. Live newborn babies with birth weights less than 2500grams were considered as cases and newborn babies with birth weight of 2500grams to 4000grams were considered as controls.

### Sample size determination and sampling technique

The sample size was calculated using Open Epi Version Two statistical software for unmatched case control with the assumption of 95% confidence level, power 80%, control to case ratio of one to two, minimum detectable odds ratio of two, and proportion of case among exposed group (birth interval less than two years) of 25.3% [16]. The final calculated sample size was 110 case and 220 controls. Both cases and controls were recruited on an ongoing basis until the required sample size was fulfilled.

### Data collection methods and tools

Five bachelor prepared nurses were trained and collected the data through face to face interviews with the post-partal mothers. The weight of the baby was collected through observation when baby is weighted using calibrated Seca scale and rounded to 100gram. Maternal mid upper arm circumference was measured using tape meter, socio demographic data, obstetric history and presence of any complication during pregnancy were collected through maternal interview. In addition, client’s medical records were reviewed for possible diagnosis of complications and gestational age. The questionnaire was initially developed in English version and translated to the local language (Amharic) for better understanding by participants. Data related to nutrition (frequency of feeding and type of diet) were collected using the Food Frequency Questionnaires (FFQs). The level of house hold food insecurity was assessed using the Household Food Insecurity Access Scale (HFIAS). This scale categorized the subjects in to four groups as secure, mild, moderate and severe insecurity [19].

### Data processing and analysis

The collected data were checked for completeness, coded, and entered into Epi Data Version 3.1 software and exported to SPSS Version 22 for analysis. Continuous data were categorized, mean and standard deviations computed, cross tabulation was done, and variables with very few frequencies were merged, when possible. The socio demographic and other profiles of the cases and control were compared using chi-square test.

Association between birth weight and independent variables were identified using bivariable and multivariable logistic regression model. In the bivariable model, variables with p-value ≤ 0.2 were selected for multivariable logistic regression model. In the multivariable logistic regression, an association was considered significant at 95% confidence level and *p*-value < 0.05. Hosmer-Lemeshow test was used to assess goodness-of-fit.

## Results

### Socio-demographic characteristics

A total of 330 women (110 cases and 220 controls) participated in this study. The minimum and maximum ages of participants were 18 and 40 years respectively with a mean and standard deviation of 26.92 ±4.69 years. Among the participants 325(98.48%) were married, 118(35.76%) were Sidama in ethnicity, and 164 (49.7 %) were Protestant. There is a significant difference among cases and controls in terms of mother’s age, place of residence, level of education, as well as presence of Refrigerator and animal breeding in the house (see Table 1).

**Table 1:** Socio-demographic characters of the mother who gave birth at Hawassa University Comprehensive Specialized Hospital, Hawassa, Ethiopia 2018

Variables	Cases (*n* = 110)	Controls (*n* = 220)	*p*-value
	Frequency (%)	Frequency (%)	
Age of Mothers			
< 24	39(35.45%)	72(32.73%)	0.001*
25–29	31(28.18%)	103(46.82%)	
> 30	40(36.36%)	45(20.45%)	
Residence			
Rural	55(50%)	57(25.91%)	0.001*
Urban	55(50%)	163(74.09%)	
Educational level			
No formal education	46(41.82%)	74(33.64%)	0.001*
Primary school	41(37.27%)	53(24.09%)	
Secondary school	12(11%)	29(13.18%)	
Above secondary school	11(10%)	64(29.09%)	
Religion			
Orthodox	12(11%)	37(16.82%)	0.243
Protestant	55(50%)	112(50.91%)	
Muslim	37(33.64%)	62(28.18%)	
Catholic	6(5.45%)	6(2.73%)	
Others^1^	0	3(1.36%)	
Ethnicity			
Sidama	40(36.36%)	78(35.45%)	0.127
Oromo	42(38.18%)	64(29.09%)	
Amhara	12(11%)	19(8.63%)	
Wolayta	7(6.36%)	25(11.36%)	
Others^2^	9(8.18%)	34(15.45%)	
Occupation			
Government Employee	28(25.45%)	72(32.73%)	0.384
Housewife	59(53.64%)	109(49.55%)	
Others3	23(20.91%)	39(17.73%)	
Has Refrigerator No	87(70.09%)	116(52.73%)	0.001
Yes	23(20.91%)	104(47.27%)	
Animal breeding			
No	66(60%)	164(74.55%)	0.007
Yes	44(40%)	56(25.45%)	
Marital status			
Married	108(98.18%)	217(98.64%)	0.75
Others4	2(1.82%)	3(1.36%)	

Others^2^: gurage, silte, gedeo, dawuro

Others^3^: student, daily laborer,

Others^4^: widowed, divorced

### Maternal obstetric and child characteristics

The minimum age at first birth was 14 years and the maximum age was 35 years with a mean and standard deviation (±SD) of 21.88 ± 3.56 years. Similarly, the minimum and maximum gestational age at first ANC visit was four weeks and 32 weeks respectively with a mean and standard deviation of 18 ± 5weeks and 2 days. In this study, 296 (89.7%) had ANC follow up during the current pregnancy and 212 (64.24 %) were multipara. The minimum and maximum birth weights of the newborns were 1000gm and 4000 gm respectively with a mean and standard deviation (±SD) of 2800±600gm. The mean gestational age at birth was 37 ± 1.8 weeks with minimum of 30 weeks and maximum of 42 weeks (see Table 2).

**Table 2:** Maternal and obstetrics characteristics of the mother who gave birth at Hawassa University Comprehensive Specialized Hospital, Hawassa, Ethiopia 2018

Variables	Cases(*n* = 110)	Controls(*n* = 220)	*p*-value
	Frequency (%)	Frequency (%)	
Gestational age (in weeks)			
< 37	41(37.27%)	53(24.1%)	0.012*
≥ 37	69(62.73%)	167(75.9%)	
Maternal Age at first birth			
< 18	40(36.36%)	35(15.91%)	0.001*
19–24	51(46.36%)	124(56.36%)	
25–35	19(17.27%)	61(27.73%)	
Parity			
Primi-para	36(32.73%)	82(37.27%)	0.417
Parous	74(67.23%)	138(62.73%)	
Birth interval in years (n = 72 case and 140 controls)			
< 2	39(54.17%)	63(45%)	0.9
≥ 2	33(45.83%)	77(55%)	
ANC during the currentpregnancy			
< 4 visits	71(64.55%)	77(35%)	0.032*
4 or more visits	39(35.45%)	143(65%)	
Dietary counseling inrecent pregnancy			
No	81(73.64%)	100(45.45%)	0.001*
Yes	29(26.36%)	120(54.55%)	
Use of iron tablets inrecent pregnancy			
No	63(57.27%)	51(23.18%)	0.001*
Yes	47(42.73%)	169(76.82%)	
Gestational Age (in months) at 1st ANC visit (case = 79, controls = 217)			
1–3	18(70.89%)	59(27.19%)	0.713
4–6	56(28.1%)	143(66%)	
7–9	5(6.33%)	15(6.91%)	
Presence of pregnancy complications			
No	67(60.91%)	185(84.09%)	0.001*
Yes	43(39.09%)	35(%15.91%)	
Sex of the newborn			
Female	46(41.82)	91(41.36)	0.885
Male	64(58.18%)	129(58.64%)	

*significant at *P*-value < 0.05

### Nutrition related characteristics of the study participants

In this study 320 (97.3%) of participants were considered household food secure and the remaining 10 were not.Factors associated with low birth weight (Table 3).

**Table 3:** Nutritional characteristics of mothers who gave birth at Hawassa University Comprehensive Specialized Hospital, Hawassa, Ethiopia 2018

Variable		Cases (%)	Controls (%)	*P*-value
Dietary counseling for this pregnancy	Yes	29(26.36%)	120(54.55%)	< 0.001*
	No	81(73.64%)	100(45.45%)	
Iron tablets use	Yes	47(42.73%)	169(76.82%)	< 0.001*
	No	63(57.27%)	51(23.18%)	
Mid upper arm circumference	≤220	35(31.82%)	27(12.27%)	< 0.001*
	> 220	75(68.18%)	193(87.73%)	
Household food security	Food secure	106(96.36%)	214(97.27%)	0.65
	Food insecure	4(3.64%)	6(2.73%)	

On binary logistic regression age of the mother, MUAC, GA, occupation, presence of complication during pregnancy, nutritional counseling, residence of the mothers, level of education, ethnicity of the mothers, age at first birth, age of the mothers, and diseases, were associated with low birth weight at the P-values of < 0.2. On multivariate logistic regression maternal MUCA less than 220mm, lack of nutritional counseling, presence of complication during pregnancy and lack of iron supplementation during pregnancy were significantly associated with LBW at p value ≤ 0.05 and 95% confidence level (Table 4).

**Table 4:** Factors associated with low birth weight among mothers who gave birth Hawassa University Comprehensive Specialized Hospital, Hawassa, Ethiopia 2018

Variables	Cases (=110)	Controls (*n* = 220)	COR (95% CI)	AOR (95% CI)	*p*-value
Age of mother					
Less than 19	40	35	0.61(0.34,1.09)	0.41(0.18,0.93)	–
20–24	51	124	0.34(0.19,0.61)	0.49(0.24, 1.05	–
30–40	19	61	1r	1r	
Gestational Age (in weeks)					
< 37	41	53	1.87(1.14,3.07)	1.79(0.98, 3.28)	–
≥ 37	69	167	1r	1r	–
Residence					
Rural	55	57	2.86(1.77, 4.62)	1.3(0.66, 2.6)	–
Urban	55	163	1r	r	
Level of Education					
Not Educated	46	74	3.62(1.73, 7.56)	0.94(0.34, 2.62)	–
Primary	41	53	4.5(2.1, 9.6)	1.72 (0.68, 4.37)	–
Secondary school	12	29	2.4 (0.95, 6.1)	1.4 (0.4, 4.6)	
Above secondary School	11	64	1r	1r	
Presence of pregnancy complications					
No	67	185	1r	1r	
Yes	43	35	3.39 (2.0, 5.74)	2.96 (1.55, 5.64)*	0.001
Presence of Refrigerator					
No	87	116	3.39 (2.0, 5.76)	1.75(.87, 3.5)	
Yes	23	104	1r	1r	
Presence of Animal breeding					
No	66	164	0.5 (0.32, 0.83)	0.7 (0.35, 1.43)	
Yes	44	56	1r	1r	
Dietary counseling during pregnancy					
No	81	100	3.35 (2.0, 5.5)	2.37 (1.3, 4.34)*	0.005
Yes	29	120	1r	1r	
Use of iron tablets during pregnancy					
No	63	51	4.4 (2.72, 7.26)	2.89 (1.58, 5.29)*	0.001
Yes	47	169	1r	1r	
Mid upper arm circumference (mm)					
< 220	35	27	3.34 (1.89,5.89)	2.9 (1.47, 5.81)*	0.002
≥ 220	75	193	1r	1r	

## Discussion

This study showed that the odds of delivering low birth weight newborns among mothers who did not get nutritional counseling during ANC were two times higher than the odds of newborns born to women who received nutritional counseling. Providing antenatal care and nutritional counseling to pregnant women is effective in increasing their dietary intake, potentiating towards a successful pregnancy and healthier pregnancy outcome [20–22]. Regular prenatal nutrition counseling increase maternal weight gain and increase birth weight of the newborns [23].

The odds of delivering low birth weight newborns among mothers whose mid upper arm circumference (MUAC) was less than 220 mm were four times higher compared to those whose MUAC was greater than 220 mm. This finding aligns with a study based on the Ethiopian Demographic Health Survey analysis, which concluded that maternal nutritional status is significantly associated with low birth weight [22]. In other studies, nutritional status was measured as extra meal utilization and presence of anemia during pregnancy where both factors were found to be determinants for low birth weight [15, 17, 22, 24]. Maternal nutritional status affect fetal growth and weight gain.

The odds of delivering low birth weight newborns among mothers with complications during pregnancy were two times higher than their counter parts. Previous studies conducted in Gondar, Bale zone, Adwa, and southern Iran also identified the presence of HIV infection, eclampsia/preeclampsia, and anemia as significantly associated with low birth weight as these predispose the fetus to intrauterine growth restrictions [11, 16, 18, 24]. It is known that any disorders that affect fetal nutritional gain during intra uterine life directly affect birth weight.

This study showed that the odds of delivering low birth weight newborns among mothers who did not use iron tablets during current pregnancy were two times higher compared to those who used iron tablets during pregnancy. Similar evidence reported in studies conducted in Addis Ababa and Adwa, where iron utilization during pregnancy was found to be protective for LBW [18, 21]. It was also supported by double blind randomized community trial study undergone in Nepal which show that iron supplementation during pregnancy increased birth weight by 37 gram on average [25]. Another randomized controlled trial study conducted in low income pregnant women in Cleveland counties showed that iron supplementation during pregnancy increase birth weight by 206 grams on average [26]. Despite its various contribution to health improvement, this study is not without limitations. One of the limitation is that since the study is conducted at a single hospital, the finding is may not be generalizable to the entire birth in the area.

Almost all factors associated with low birth weight in this study were preventable and can be controlled easily. Strengthening the integration of nutrition counseling in to ANC help could to improve maternal nutritional status during pregnancy [27]. Even though around 90% of participants have ANC follow up, only 43% of the cases used iron. During ANC visit giving emphasis on counselling on the importance of iron use can improve the number of users and improve birth weight.

## Conclusions

Lack of nutritional counseling during pregnancy, , maternal mid upper arm circumference less than 220mm, and presence of complication during pregnancy were significantly associated with low birth weight. Focusing on nutritional counseling and adherence to iron supplementations need great attention from care providers during prenatal care.

## Abbreviations

ANC: Antenatal care
EDHS: Ethiopian Demographic and health survey
FFQ: Food frequency questionnaire
HFIAS: Household food insecurity access scale
LBW: Low birthweight
MUAC: Mid-upper arm circumstance
SPSS: Software package for social sciences
UNICEF: United Nations Children’s Fund
WHO: World Health Organization
